# Triclosan-induced genes *Rv1686c*-*Rv1687c* and *Rv3161c* are not involved in triclosan resistance in *Mycobacterium tuberculosis*

**DOI:** 10.1038/srep26221

**Published:** 2016-05-19

**Authors:** Andromeda Gomez, Núria Andreu, Mario Ferrer-Navarro, Daniel Yero, Isidre Gibert

**Affiliations:** 1 Institut de Biotecnologia i de Biomedicina (IBB), Universitat Autònoma de Barcelona (UAB), 08193 Bellaterra (Cerdanyola del Vallès), Barcelona, Spain; 2 Departament de Genètica i de Microbiologia, Universitat Autònoma de Barcelona (UAB), 08193 Bellaterra (Cerdanyola del Vallès), Barcelona, Spain

## Abstract

A key issue towards developing new chemotherapeutic approaches to fight *Mycobacterium tuberculosis* is to understand the mechanisms underlying drug resistance. Previous studies have shown that genes *Rv1686c-Rv1687c* and *Rv3161c*, predicted to encode an ATP-binding cassette transporter and a dioxygenase respectively, are induced in the presence of triclosan and other antimicrobial compounds. Therefore a possible role in drug resistance has been suggested for the products of these genes although no functional studies have been done. The aim of the present study was to clarify the role of Rv1686c-Rv1687c and Rv3161c in *M. tuberculosis* resistance to triclosan and other drugs. To this end, deficient mutants and overproducing strains for both systems were constructed and their minimal inhibitory concentration (MIC) against over 20 compounds, including triclosan, was evaluated. Unexpectedly, no differences between the MIC of these strains and the wild-type H37Rv were observed for any of the compounds tested. Moreover the MIC of triclosan was not affected by efflux pump inhibitors that inhibit the activity of transporters similar to the one encoded by *Rv1686c-Rv1687c*. These results suggest that none of the two systems is directly involved in *M. tuberculosis* resistance to triclosan or to any of the antimicrobials tested.

Tuberculosis remains one of the most important infectious diseases worldwide, killing more than 1.5 million people each year^1^. New drugs are urgently needed to shorten the duration of the current treatment, and to treat *Mycobacterium tuberculosis* strains resistant to existing antibiotics. The unusual mycobacterial cell wall constitutes a proved target for the development of new antimycobacterial drugs (for a review, see reference 2). In fact, the current front line treatment regimen relies on isoniazid (INH), a drug that compromises the integrity of the cell wall by inhibiting the biosynthesis of mycolic acids. Specifically, INH inhibits the enoyl acyl carrier protein reductase InhA, upon activation by the mycobacterial catalase-peroxidase enzyme KatG^3^,^4^,^5^,^6^. Most clinical isolates resistant to INH carry mutations in *katG*^7^, therefore compounds that inhibit InhA but that do not require activation by KatG, have great promise as novel drugs for combating drug resistant strains.

One such compound is triclosan, a biocide with a high antibacterial activity used as an ingredient of diverse products like soaps, detergents, toothpastes, dishwashing liquids and lotions, among others^8^. Triclosan inhibits InhA from *M. tuberculosis* without the need for prior activation^9^,^10^,^11^. However, triclosan is less potent than INH and thus must be used at higher concentrations, causing broader disruption of bacterial cell wall functions and upregulation of many genes encoding transport proteins and membrane-bound proteins in *M. tuberculosis* and in many other bacteria^12^,^13^,^14^,^15^. Nevertheless, triclosan has been used as a starting point for structure-based development of a series of alkyl diphenyl ethers that are stronger inhibitors of InhA and can prevent growth of both sensitive and INH-resistant strains^16^,^17^,^18^,^19^.

Betts and collaborators studied the transcriptional response of *M. tuberculosis* to triclosan in order to better understand its mechanism of action^12^. This study exposed two possible detoxification systems that were highly induced after treatment with triclosan: an ABC transporter encoded by *Rv1686c-Rv1687c*, similar to known antibiotic resistance systems, and a dioxygenase encoded by *Rv3161c* with similarity to several bacterial aromatic dioxygenases. It was suggested that the product of these genes could be involved in *M. tuberculosis* resistance to triclosan. The fact that the more active triclosan derivatives failed to induce these genes was proposed to contribute to their improved whole-cell activity compared to triclosan^16^,^17^.

Both sets of genes are also induced in response to treatment with compounds such as carbonyl cyanide *m*-chlorophenylhydrazone (CCCP), the antitubercular ARP4, or 2,4-dinitrophenol^13^. Genes *Rv1686c-Rv1687c* are also induced by lupulone, a compound extracted from hops (*Hurnulus lupulus*) that exhibits a good activity against *M. tuberculosis*^20^. *Rv3161c* is also induced by thioridazine^21^ and compounds SRI#967 and SRI#9190^22^, all of which contain benzene ring structures like triclosan. It has therefore been proposed that the putative dioxygenase Rv3161c could hidroxylate benzenes, thus being partly responsible for *M. tuberculosis* natural resistance to them^22^.

Despite all the data suggesting the involvement of these genes in *M. tuberculosis* drug resistance, to our knowledge, no functional studies have been reported. A better understanding of *M. tuberculosis* detoxification mechanisms is important in the development of new drugs since they are relevant both in natural resistance and in acquired resistance. For example, overexpression of detoxification mechanisms such as efflux pumps and degradation or modification enzymes could lead to a low-level resistance favouring the acquisition of chromosomal mutations conferring higher levels of drug resistance^23^,^24^,^25^. Therefore the aim of the present study was to clarify the role of Rv1686c-Rv1687c and Rv3161c in *M. tuberculosis* resistance to triclosan and other drugs. Using mutant and overexpressing strains for each system we have found that these genes are not necessary for resistance to triclosan or to any of the other compounds here tested.

## Results and Discussion

A previous transcriptional study of *M. tuberculosis* response to triclosan showed that genes *Rv1686c-Rv1687c* and *Rv3161c* are highly induced, and a possible role of the products of these genes in triclosan detoxification was suggested^12^,^16^. In order to clarify the potential involvement of these genes in drug resistance, firstly, we studied expression of *Rv1687c* and *Rv3161c* by qRT-PCR after exposing the wild-type *M. tuberculosis* H37Rv strain to triclosan (5× MIC) or to DMSO (untreated control) for 2 hours so as to reproduce the approach previously described by others^12^. Our results showed that triclosan leads to a 1002.67 (1001.84–1003.49) and a 1366.83 (1366.36–1367.30) fold increase in the expression of *Rv1687c* and *Rv3161c*, respectively, confirming that triclosan strongly induces these systems. In fact, the induction that we observed was 5 times higher than that previously described. This is probably due to the higher concentration of triclosan that we used since the MIC for triclosan that we obtained was also higher (21.7 μg/mL vs 8 μg/mL^12^ or 12.5 μg/mL^16^).

**Figure 1:**
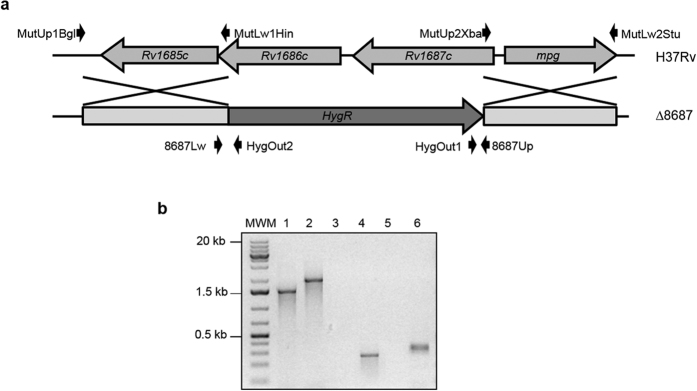
Construction and confirmation of Δ8687. (**a**) Physical map of the region containing genes *Rv1686c-Rv1687c* depicting primers used to construct and confirm the knock-out strain. (**b**) Confirmation PCRs. Lane 1: H37Rv and lane 2: Δ8687, amplified with primers 8687Up and 8687Lw (1.5 and 2 kb, respectively); lane 3: H37Rv and lane 4: Δ8687, amplified with HygOut1 and 8687Up (no amplification and 285 pb, respectively); lane 5: H37Rv and lane 6: Δ8687, amplified with HygOut2 and 8687Lw (no amplification and 358 pb, respectively). MWM: molecular weight marker.

**Figure 2:**
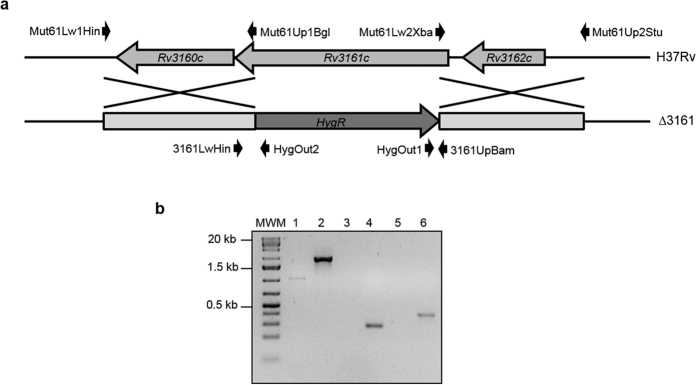
Construction and confirmation of Δ3161. (**a**) Physical map of the region containing gene *Rv3161c* depicting primers used to construct and confirm the knock-out strain. (**b**) Confirmation PCRs. Lane 1: H37Rv and lane 2: Δ3161, amplified with primers 3161UpBam and 3161LwHin (1.1 and 2 kb, respectively); lane 3: H37Rv and lane 4: Δ3161, amplified with HygOut1 and 3161UpBam (no amplification and 310 bp, respectively); lane 5: H37Rv and lane 6: Δ3161, amplified with HygOut2 and 3161LwHin (no amplification and 354 bp, respectively). MWM: molecular weight marker.

Once the induction of these genes was confirmed, we constructed *M. tuberculosis* H37Rv knockout mutant strains for each system using phage-mediated allelic exchange. To construct the knockout strain of *Rv1686c-Rv1687c,* named Δ8687, 608 bp out of 681 bp of *Rv1686c* and 730 bp out of 768 bp of *Rv1687c* were deleted and a hygromycin cassette inserted (Fig. 1). Likewise, to construct Δ*Rv3161c,* 991 bp out of 1149 bp were deleted and a hygromycin cassette inserted (Fig. 2). Allelic exchange was confirmed by PCR using primers specific to the hygromycin cassette and to the genomic flanking regions. PCR products would only be obtained if the hygromycin cassette had inserted into the correct location on the chromosome. PCR products of the expected size were obtained for the knockout strains; no products were obtained for the wild-type strain (Figs 1 and 2). PCR over the region of disruption further confirmed mutant construction by showing an increase in size corresponding to insertion of the hygromycin cassette at this location. These PCRs also allowed discriminating single from double recombinants.

**Figure 3:**
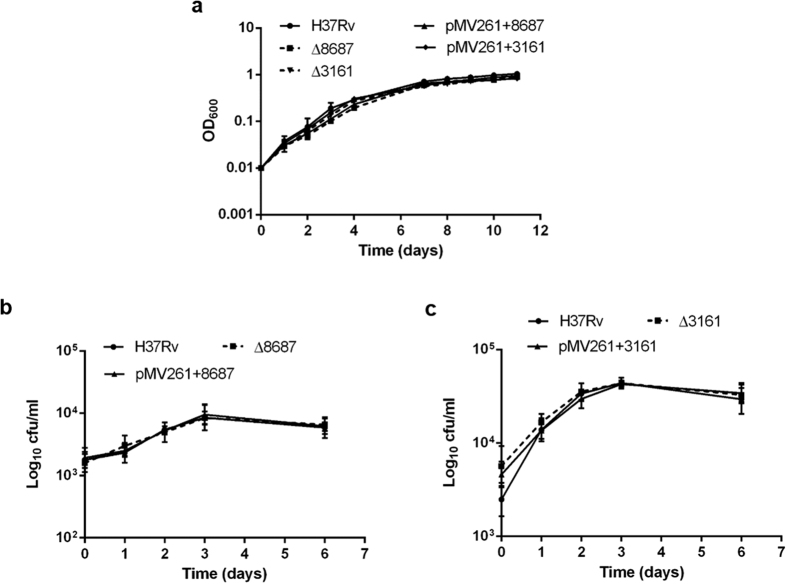
Growth of the parental, mutant and overexpressing strains in (**a**) Middlebrook 7H9 supplemented with 0.05% Tween 80 and 10% ADC, and (**b**,**c**) inside J774 macrophages. Results are expressed as the average and standard deviation of duplicates of at least two independent experiments.

We also constructed *M. tuberculosis* overexpressing strains for each system, using the replicative expression vector pMV261 with the strong promoter from gene *hsp60*, resulting in strains H37Rv (pMV261 + 8687) and H37Rv (pMV261 + 3161). Overexpression of *Rv1687c* and *Rv3161c* was confirmed measuring gene expression by qRT-PCR (6.71 and 9.39, and 127.96 and 160.67 fold increase, respectively, for two independent clones of each strain), and the overproduced proteins were confirmed by 2D-PAGE and MALDI-TOF MS/MS (see Supplementary Figs S1 and S2). Neither the mutant nor the overexpressing strains presented any evident phenotypes in terms of colony morphology, *in vitro* growth or growth inside macrophages (Fig. 3).

We then determined the MICs of over 20 compounds including antibiotics, dyes and biocides against the wild-type parental strain *M. tuberculosis* H37Rv, the control strain pMV261 (containing vector pMV261), three independent clones of each knockout mutant strain, and two independent clones of each overexpressing strain. Unexpectedly, no significant differences in the MICs for any of the tested compounds, including triclosan, were observed (Table 1). These results are inconsistent with a possible role for these genes in resistance to triclosan or to the other drugs tested.

To further confirm these results we assessed the effect of efflux pump inhibitors (reserpine, CCCP, verapamil and *o*-vanadate) on the MIC of triclosan in the wild-type H37Rv strain. These compounds inhibit the activity of transporters including ABC transporters that are similar to the one encoded by *Rv1686c-Rv1687c*. First, we determined the MIC of the inhibitors in H37Rv (reserpine: 48 μg/mL, CCCP: 5 μg/mL, verapamil: 80 μg/mL and *o*-vanadate: >96 μg/mL). Then, we evaluated the effect of the efflux pump inhibitors on the MIC of triclosan, using the inhibitors at subinhibitory concentrations to ensure that the cellular viability was not affected (reserpine: 12 μg/mL, CCCP: 1.25 μg/mL, verapamil: 20 μg/mL and *o*-vanadate: 9 μg/mL). No changes were observed in the MIC of triclosan with any of the inhibitors tested. Therefore, with our experimental procedure, we did not find any evidence supporting the implication of efflux pumps such as Rv1686c-Rv1687c in mediating triclosan resistance in *M. tuberculosis*.

Altogether the results presented here strongly suggest that the products of genes *Rv1686c-Rv1687c* and *Rv3161c* are not involved in resistance to triclosan or to any of the other compounds tested in *M. tuberculosis*. It could be speculated that induction of these genes in response to triclosan is part of an unsuccessful attempt of the bacteria to counteract the nonspecific toxic effects caused by triclosan when used at high concentrations, such as the ones needed to kill *M. tuberculosis* because of its moderate inhibitory activity against its target InhA. This would also explain the non-induction of *Rv1686c-Rv1687c* and *Rv3161c* by the more active triclosan-derivatives as they have an increased potency towards InhA resulting in a more narrowed mode of action and a lower MIC^17^. Although transcriptional studies are important and shed light onto many aspects of the mechanism of action and bacterial response to drugs, the results obtained here highlight the importance of corroborating transcriptional data with functional studies.

## Methods

### Bacterial strains, plasmids, media and growth conditions

Strains and plasmids used are listed in Supplementary Table S1. *M. tuberculosis* and *M. smegmatis* were grown in Middlebrook 7H9 supplemented with 0.05% Tween 80 and 10% albumin-dextrose-catalase (ADC), or on 7H10 agar supplemented with 10% oleic acid-albumin-dextrose-catalase (OADC). *Escherichia coli* was grown in Luria-Bertani (LB) medium. The following antibiotics were used when required: hygromycin B (100 μg/mL for mycobacteria and *E. coli*), kanamycin (25 μg/mL for mycobacteria, 50 μg/mL for *E. coli*), and ampicillin (50 μg/mL for *E. coli*). All cultures were incubated at 37 °C.

### Quantitative Real-time PCR

Total RNA was extracted from 30 mL of *M. tuberculosis* mid-log cultures in triplicate using the GTC/Trizol method^26^,^27^. The RNA was cleaned with 75% ethanol and purified using an RNeasy Minikit (Qiagen). RNAs were treated with Turbo DNAse (Ambion, Life Technologies) and DNA contamination was checked by PCR. Quality and quantity of RNA were determined using a Bio-analyser (Agilent).

cDNA was synthesized using the high capacity cDNA reverse transcription kit (Applied Biosystems). Primers and TaqMan probes were designed by Applied Biosystems for *Rv1687c, Rv3161c* and *sigA* genes (Supplementary Table S2). qRT-PCR was performed on an Applied Biosystems 7500 Real-Time System using TaqMan universal PCR master mix (Applied Biosystems). Linear amplification and amplification efficiencies for each TaqMan primer/probe were determined. Real-time analysis was performed in quadruplicate on RNA from three independent cultures. Quantification of *sigA* expression served as an internal control. Fold change was calculated as a ratio of the arbitrary expression units, standardised to *sigA*^28^.

### Construction of mutant strains

Phage-mediated allelic exchange was used to create the *M. tuberculosis* Δ*Rv1686c*-*Rv1687c* (Δ*8687*) and Δ*Rv3161c* mutant strains as previously described^29^. Upstream and downstream flanking sequences from genes *Rv1686c-Rv1687c* and *Rv3161c* (Figs 1 and 2) were amplified using primers listed in Supplementary Table S2. The flanking regions were cloned in pGEM-T^®^ (Promega), verified by sequencing and cloned into pYUB854 flanking the hygromycin cassette. The obtained plasmids pYUB854Δ*8687* and pYUB854Δ*Rv3161c* were digested with PacI, cloned in the temperature-sensitive shuttle phasmid phAE159, packaged using MaxPlax Kit (Epicenter) and transduced in *E. coli* HB101 according to the manufacturer’s instructions. Recombinant phasmids were verified by PacI digestion and PCR, and were electroporated in *M. smegmatis* mc^2^ 155 as previously described^30^. A high titer lysate prepared at the permissive temperature (30 °C) was used to transduce *M. tuberculosis* at the non-permissive temperature (37 °C). Transductants were selected on hygromycin and successful deletion was confirmed by PCR using primers that anneal to the flanking regions and hygromycin cassette specific primers (Figs 1 and 2, and Supplementary Table S2).

### Construction of overexpressing strains

Genes *Rv1686c*-*Rv1687c* and *Rv3161c* were amplified by PCR using primers described in Supplementary Table S2, and cloned into pMV261 using BalI-EcoRI and BamHI–HindIII, respectively. The resulting plasmids pMV261 + 8687 and pMV261 + 3161 were verified by sequencing and electroporated into *M. tuberculosis* H37Rv as previously described^30^. The recombinant strains were checked by recovering the plasmid after transformation into *E. coli*.

### Two-Dimensional Electrophoresis and Protein identification

Protein extracts were obtained from 140 mL of *M. tuberculosis* mid-log cultures grown in 7H9–0.05% Tween 80 supplemented with 10% ADC. Cells were pelleted by centrifugation and washed twice with sterile PBS 1X. Pellets were resuspended in lysis solution (7M Urea, 2 M Thiourea, 4% CHAPS, 4% ASB-14) and then sonicated (20 cycles: 30 sec ON/1 min OFF) at 4 °C in ice water, and finally centrifuged to remove cells debris. Samples were then quantified and purified using 2-D Quant Kit and 2-D Clean-Up Kit (GE Healthcare) respectively. For two-dimensional gel electrophoresis (2DE) IPGphor II and EttanSix (GE Healthcare) were used for the first and second dimension respectively. 100 μg of each sample was separated by 2DE in 24 cm length, 6–9 pH strips in the first dimension and in a 12% SDS-PAGE for the second dimension according to manufacturer instructions. 2DE gels were silver stained as described elsewhere^31^. Image analysis was performed using Progenesis SameSpot software (Nonlinear Dynamics). After image analysis, differential spot was excised and identified as previously described by mass spectrometry^32^.

### Growth analysis of strains

*M. tuberculosis* wild type, mutant and overexpressing strains were grown to mid-log phase (OD_600_ 0.8–1) in 7H9 media as previously stated. The cells were then used to inoculate fresh medium to a starting OD_600_ of 0.01. Growth was analysed by taking daily OD_600_ readings.

### *In vitro* infection of J774 murine macrophages

J 774 cells were maintained in Dulbecco’s minimal essential medium (DMEM) supplemented with 10% heat-inactivated fetal bovine serum. The cells were plated at a concentration of 5 × 10^4^ cells per well in 96-well tissue culture plates with clear bottoms (Corning^®^) and allowed to adhere overnight. For the infection, mid-log phase *M. tuberculosis* were washed twice with PBS + 0.05% Tween, once with Dulbecco’s PBS and then allowed to stand for 5 min, before the supernatant were collected. The bacteria were then diluted in DMEM and added to the J 774 cells at a concentration of ∼5 × 10^5 ^cfu/well. After 4 h of infection at 37 °C in 5% CO_2_, macrophages were treated with 200 mg/L amikacin for 1 h and washed twice with PBS to eliminate any extracellular bacteria. Lastly, 200 μL of complete DMEM was added to each well. Intracellular survival and growth was assessed by lysis of the monolayers by the addition of water followed by a 30 min incubation at room temperature and enumeration of bacteria by serial dilution in PBS-Tween plating onto Middlebrook 7H10 solid medium. Colonies were counted after 3–4 weeks incubation at 37  °C and the average cfu/well determined.

### Antimicrobials agents and inhibitors

Triclosan, INH, ethambutol, rifampicin, clofazimine, ethionamide, *p*-aminosalicylic acid, D-cycloserine, tetracycline, erythromycin, gentamicin, ofloxacin, levofloxacin, acriflavine, thioridazine hydrochloride, reserpine, *o*-vanadate, CCCP, verapamil, and cetyltrimethyl ammonium chloride (CTAB) were obtained from SIGMA. Streptomycin, amikacin, and kanamycin were obtained from Apollo Chemicals Ltd. Chloramphenicol was obtained from Boehringer Mannheim, hygromycin B from Roche, safranin from Panreac, and ethidium bromide from Amresco. Stocks were prepared according to the manufacturer’s instructions, and aliquots kept at −20 °C were thawed just before use. For the MIC assays, drugs were diluted to the working concentration in Middlebrook 7H9 supplemented with 0.2% glycerol and 10% ADC.

### MIC determination

MICs were determined by the resazurin microtitre assay (REMA) as previously described^33^. Briefly, mid-log phase *M. tuberculosis* cultures were diluted in 7H9 supplemented with 10% ADC and 0.2% glycerol to a final OD_600_ of 0.01. Aliquots of 100 μL were then added to each well of a 96-well plate containing 100 μL of 2-fold serial dilutions of each compound in duplicate. Sterility (no bacteria) and growth (no antibiotic) controls were also prepared. 30 μL of resazurin (0.01%) were added to the growth control wells after 7 days of incubation at 37 °C, and the plates were incubated for additional 24 h. If a colour change from blue (oxidized state) to pink (reduced state), indicating bacterial growth, was observed, resazurin was added to the remaining wells and the plates were incubated at 37 °C for additional 24 h. The visual MIC was determined as the lowest antibiotic concentration which prevented colour change. The MIC of triclosan was also determined in combination with the efflux pump inhibitors CCCP (1.25 μg/mL), reserpine (12 μg/mL), verapamil (20 μg/mL) and *o*-vanadate (9 μg/mL).

### Statistical analysis

Statistical analyses were performed using GraphPad Prism 5.02 (GraphPad Software, San Diego, USA). Normality of data was tested by use of the D’Agostino & Pearson omnibus normality test. According to this, differences were assessed by use of the non-parametric Mann-Whitney test, or the t test for normal data. The difference was considered to be statistically significant when *p* < 0.05.

## Additional Information

**How to cite this article**: Gomez, A. *et al*. Triclosan-induced genes *Rv1686c-Rv1687c* and *Rv3161c* are not involved in triclosan resistance in *Mycobacterium tuberculosis. Sci. Rep.* **6**, 26221; doi: 10.1038/srep26221 (2016).

## Supplementary Material

Supplementary Information

## Figures and Tables

**Table 1:** MICs (μg/mL) of several antimicrobial compounds against *M. tuberculosis* H37Rv, pMV261, and the *Rv1686c-Rv1687c* and *Rv3161c* mutant and overexpressing strains.

Compound	*M. tuberculosis* strain					
	H37Rv	pMV261	pMV261 + 8687	Δ8687	pMV261 + 3161	Δ3161
Triclosan	21.7 (21.7)	21.7 (21.7)	21.7 (21.7)	21.7 (21.7)	21.7 (21.7)	21.7 (21.7)
INH	0.062 (0.062–0.12)	0.12 (0.062–0.12)	0.12 (0.062–0.12)	0.062 (0.062–0.12)	0.062 (0.062)	0.093 (0.062–0.12)
Ethambutol	1 (1)	1 (1)	1 (1)	1 (1)	1 (1)	1 (1)
Streptomycin	0.125 (0.125)	0.125 (0.125)	0.125 (0.125)	0.125 (0.125)	0.125 (0.125)	0.125 (0.125)
Rifampicin	0.064 (0.016–0.064)	0.064 (0.032–0.064)	0.064 (0.032–0.064)	0.064 (0.016–0.064)	0.064 (0.064)	0.064 (0.032–0.064)
Clofazimine	0.12 (0.12–0.24)	0.12 (0.12–0.24)	0.12 (0.12)	0.12 (0.12)	0.12 (0.12–0.24)	0.12 (0.12–0.24)
Ethionamide	0.48 (0.24–0.48)	0.48 (0.24–0.48)	0.48 (0.48)	0.48 (0.48)	0.48 (0.24–0.48)	0.48 (0.24–0.48)
*p*–aminosalicylic acid	0.1 (0.1–0.2)	0.1 (0.1–0.2)	0.1 (0.1–0.2)	0.1 (0.1)	0.1 (0.1–0.2)	0.1 (0.1–0.2)
D–cycloserine	8 (8)	8 (8)	8 (8)	8 (8)	8 (8)	8 (8)
Kanamycin	2 (2)	n.d.	n.d.	2 (2)	n.d.	2 (2)
Tetracycline	4 (2–8)	4 (4–8)	4 (4–8)	4 (4)	4 (4–8)	4 (4)
Erythromycin	100 (50–100)	100 (50–100)	100 (50–100)	100 (50–100)	100 (100)	100 (50–100)
Chloramphenicol	2.5 (2.5)	2.5 (2.5)	2.5 (2.5)	2.5 (2.5)	2.5 (2.5)	2.5 (2.5)
Gentamycin	3 (3)	3 (3)	3 (3)	3 (3)	3 (3)	3 (3)
Ofloxacin	0.25 (0.25)	0.25 (0.25)	0.25 (0.25)	0.25 (0.25)	0.25 (0.25)	0.25 (0.25)
Levofloxacin	0.125 (0.125)	0.125 (0.125)	0.125 (0.125)	0.125 (0.125)	0.125 (0.125)	0.125 (0.125)
Acriflavin	2 (2)	2 (2)	2 (2)	2 (2)	2 (2)	2 (2)
Thioridazine hydrochloride	10 (5–10)	10 (5–10)	10 (10)	10 (10)	10 (10)	10 (10)
Amikacin	0.5 (0.5–1)	1 (1)	0.5 (0.5)	0.5 (0.5)	0.75 (0.5–1)	0.5 (0.5)
Ethidium bromide	2 (2)	1 (1–2)	2 (2)	1.5 (1–2)	2 (2)	1.5 (1–2)
Safranin	1 (1)	1 (1)	1 (1)	1 (1)	1 (1)	1 (1)
CTAB	25 (25)	25 (25)	25 (25)	25 (25)	25 (25)	25 (25)

Results are expressed as the median and range (minimum-maximum) of duplicates of at least two independent experiments. Two independent clones of the overexpressing strains and three of the mutant strains were tested with similar results.

n. d., non determined since pMV261 contains a kanamycin resistance selection marker.
