# Supplementary material for: Triclosan-induced genes Rv1686c-Rv1687c and Rv3161c are not involved in triclosan resistance in Mycobacterium tuberculosis
Source: Sci Rep. 2016 May 19;6:26221. doi: 10.1038/srep26221 (PMC4872132; doi:10.1038/srep26221)
Supplement: Supplementary Information [file srep26221-s1.pdf]

Triclosan-induced genes *Rv1686c-Rv1687c* and *Rv3161c* are not involved in triclosan resistance in *Mycobacterium tuberculosis*

Andromeda Gómez[1,2,¥], Núria Andreu[1,*,§], Mario Ferrer-Navarro[1,#], Daniel Yero[1] and Isidre Gibert[1,2,*]

[1]Institut de Biotecnologia i de Biomedicina (IBB), Universitat Autònoma de Barcelona (UAB), 08193 Bellaterra (Cerdanyola del Vallès), Barcelona, Spain

[2]Departament de Genètica i de Microbiologia, Universitat Autònoma de Barcelona (UAB), 08193 Bellaterra (Cerdanyola del Vallès), Barcelona, Spain.

*Corresponding authors: nuria.andreum@gmail.com, Isidre.Gibert@uab.cat

[¥]Present address: Servei de Microbiologia. Hospital Universitari Germans Trias i Pujol. Institut d'Investigació Germans Trias i Pujol. Carretera del Canyet s/n, 08916 Badalona, Spain

[§]Present address: Pathogen Molecular Biology Department, Faculty of Infectious and Tropical Diseases, London School of Hygiene and Tropical Medicine, Keppel St., London WC1E 7HT, UK

[#]Present address: Centre de Recerca en Salut Internacional de Barcelona, C/Rosselló 132, 08036 Barcelona, Spain.

Supplementary Information

Supplementary Table S1. Strains and plasmids used in this study.

Strains / Plasmids	Description	Source / Reference
***M. tuberculosis* strains:**		
H37Rv (ATCC 27294)	Wild-type	Laboratory collection
pMV261	H37Rv containing pMV261	This study
Δ8687	H37Rv knock out for *Rv1686c* and *Rv1687c*	This study
Δ3161	H37Rv knock out for *Rv3161c*	This study
H37Rv (pMV261+8687)	H37Rv containing pMV261+8687	This study
H37Rv (pMV261+3161)	H37Rv containing pMV261+3161	This study
***M. smegmatis* strains**		
mc^2155	Wild-type	Laboratory collection
***E. coli* strains**		
DH5α	*recA1 endA1 hsdR17 gyrA96 supE44 thi*-1 *relA1*Δ(*lacZYA-argF*)*U169 deoR* F80lacZΔM15	Laboratory collection
HB101	*F-supE44 Δ(mcrC-mrr) recA13 ara-14 proA2leuB6 lacY1 galk2 rpsl20(StrR) xyl-5 mtl-1 thi-1*	Laboratory collection
**Plasmids:**		
pMV261	Replicative expression vector with mycobacterial hsp60 promoter, Km^r, *oriE*, *oriM*	1
pYUB854	*E. coli* vector used for cloning allelic exchange substrate. Contains Hyg^r cassette flanked by MCS and γδ res sites, *oriE*, and λ-cos packaging site	2
phAE159	Temperature-sensitive shuttle phasmid derived from TM4, Km^r.	3
pMV261+8687	pMV261 expressing genes *Rv1686c-Rv1687c*	This study
pMV261+3161	pMV261 expressing gene *Rv3161c*	This study
pYUB854Δ8687	pYUB854 containing the DNA regions flanking *Rv1686c-Rv1687c*	This study
pYUB854Δ3161	pYUB854 containing the DNA regions flanking *Rv3161c*	This study
phAE159Δ8687	phAE159 containing pYUB854Δ8687	This study
phAE159Δ3161	phAE159 containing pYUB854Δ3161	This study

Supplementary Table S2. Primers used in this study.

Primers	Sequence (5'-3')	Purpose
Rv1687c_F	GCAAGCCGCCGATGAG	Taqman forward primer for qRT-PCR
Rv1687c_R	CCGGACAGGTTGGCACAA	Taqman reverse primer for qRT-PCR
Rv1687c_M	CCACCGCTTCGATCAC	FAM reporter probe for qRT-PCR
Rv3161c_F	CGAATTCGCCGGCTATCG	Taqman forward primer for qRT-PCR
Rv3161c_R	TCCAATTAGCTCGCCACTCATG	Taqman reverse primer for qRT-PCR
Rv3161c_M	CTCGACCTGCACCATC	FAM reporter probe for qRT-PCR
SigA_F	CCGCCCGACCGATAGC	Taqman forward primer for qRT-PCR
SigA_R	ATGTCGAATGTCGGCGTTGATA	Taqman reverse primer for qRT-PCR
SigA_M	TCAGCGTGAATACTCG	FAM report probe for qRT-PCR
Mut61Up2Stu	AGGCCTGTTGCTGTCGACGTTCTTGA	Cloning of *Rv3161c* upstream region into pYUB854 for mutant construction
Mut61Lw2Xba	TCTAGATAATCCCCGATGTCGGTAAG	
Mut61Up1Bgl	AGATCTGAACGGGGTCACCTTAGCTC	Cloning of *Rv3161c* downstream region into pYUB854 for mutant construction
Mut61Lw1Hin	AAGCTTGAAAATTCTGGTTCCCGTCA	
MutUp2Xba	TCTAGACCGTCGCGAAGTAATTCAT	Cloning of *Rv1686c-Rv1687c* upstream region into pYUB854 for mutant construction
MutLw2Stu	AGGCCTGAGCTCATCGAGGATCATGC	
MutLw1Hin	AAGCTTCGTCGTCGTGCTGAGTTTC	Cloning of *Rv1686c-Rv1687c* downstream region into pYUB854 for mutant construction
MutUp1Bgl	AGATCTGCGACATCCAGGTGCTCTAT	
8687Up	TGGCCATGATGATTTCATCAAGTG	Cloning of *Rv1686c-Rv1687c* into pMV261, and confirmation of Δ8687
8687Lw	GAATTCTATGACGTCCGTCGCCG	Cloning of *Rv1686c-Rv1687c* into pMV261, and confirmation of Δ8687
3161UpBam	GGATCCGATGTTATCAACTGATAAC	Cloning of *Rv3161c* into pMV261, and confirmation of Δ3161
3161LwHin	AAGCTTCTAGCTGGCACCTGGGTG	Cloning of *Rv3161c* into pMV261, and confirmation of Δ3161
HygOut1	GCATGCAAGCTCAGGATGTC	Confirmation of Δ8687 and Δ3161
HygOut2	TTCGAGGTGTTCGAGGAGAC	Confirmation of Δ8687 and Δ3161

*Restrictions sites used for cloning are underlined

Supplementary Figure S1. Proteomics analysis of the overexpressing strain H37Rv (pMV261+8687). **(a, b)** Representative image of the 2DE gels (pH 6-9) of strain H37Rv containing the empty vector pMV261 and of the overexpressing strain H37Rv (pMV261+8687), respectively.**(c)** MALDI-TOF spectra of the spot indicated by an arrow in **(b)**, each peak matches with a peptide from tryptic digestion of the protein. **(d)** Amino acid sequence alignment of Rv1687c from the TubercuList database and Rv1687c obtained by MALDI-TOF MS. Black highlighting indicates identical residues.

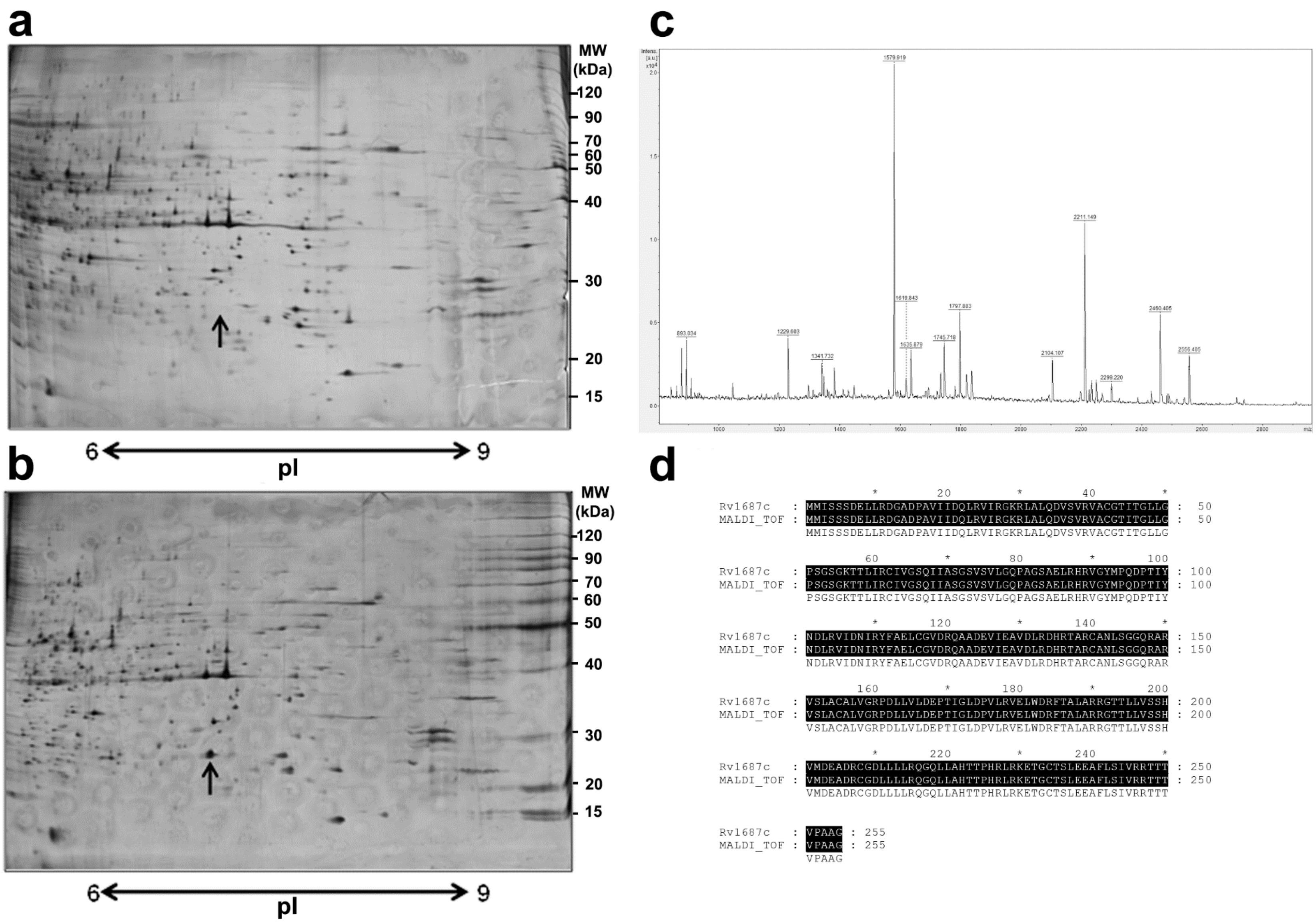
a
MW (kDa)
120
90
70
60
50
40
30
20
15
6
9
pI
b
MW (kDa)
120
90
70
60
50
40
30
20
15
6
9
pI
c
Intens. [a.u.]
x10^4
2.0
1.5
1.0
0.5
0.0
1579.919
2211.149
1619.843
1797.883
2460.405
893.034
1229.603
1745.718
1635.879
1341.732
2104.107
2556.405
2299.220
1000
1200
1400
1600
1800
2000
2200
2400
2600
2800
m/z
d
* 20 * 40 *
Rv1687c : MMISSSDELLRDGADPAVIIDQLRVIRGKRLALQDVSVRVACGTITGLLG : 50
MALDI_TOF : MMISSSDELLRDGADPAVIIDQLRVIRGKRLALQDVSVRVACGTITGLLG : 50
MMISSSDELLRDGADPAVIIDQLRVIRGKRLALQDVSVRVACGTITGLLG
60 * 80 * 100
Rv1687c : PSGSGKTTLIRCIVGSQIIASGSVSVLGQPAGSAELRHRVGYMPQDPTIY : 100
MALDI_TOF : PSGSGKTTLIRCIVGSQIIASGSVSVLGQPAGSAELRHRVGYMPQDPTIY : 100
PSGSGKTTLIRCIVGSQIIASGSVSVLGQPAGSAELRHRVGYMPQDPTIY
* 120 * 140 *
Rv1687c : NDLRVIDNIRYFAELCGVDRQAADEVIEAVDLRDHRTARCANLSGGQRAR : 150
MALDI_TOF : NDLRVIDNIRYFAELCGVDRQAADEVIEAVDLRDHRTARCANLSGGQRAR : 150
NDLRVIDNIRYFAELCGVDRQAADEVIEAVDLRDHRTARCANLSGGQRAR
160 * 180 * 200
Rv1687c : VSLACALVGRPDLLVLDEPTIGLDPVLRVELWDRFTALARRGTTLLVSSH : 200
MALDI_TOF : VSLACALVGRPDLLVLDEPTIGLDPVLRVELWDRFTALARRGTTLLVSSH : 200
VSLACALVGRPDLLVLDEPTIGLDPVLRVELWDRFTALARRGTTLLVSSH
* 220 * 240 *
Rv1687c : VMDEADRCGDLLLLRQGQLLAHTTPHRLRKETGCTSLEEAFLSIVRRTTT : 250
MALDI_TOF : VMDEADRCGDLLLLRQGQLLAHTTPHRLRKETGCTSLEEAFLSIVRRTTT : 250
VMDEADRCGDLLLLRQGQLLAHTTPHRLRKETGCTSLEEAFLSIVRRTTT
Rv1687c : VPAAG : 255
MALDI_TOF : VPAAG : 255
VPAAG

Supplementary Figure S2. Proteomics analysis of the overexpressing strain H37Rv (pMV261+3161). **(a, b)** Representative image of the 2DE gels (pH 4-7) of strain H37Rv containing the empty vector pMV261 and of the overexpressing strain H37Rv (pMV261+3161), respectively. A 3D view of the highlighted square region for each gel has been included to facilitate the identification of the spot. **(c)** MALDI-TOF spectra of the spot indicated by arrow in **(b)**, each peak matches with a peptide from tryptic digestion of the protein. **(d)** Amino acid sequence alignment of Rv3161c from the TubercuList database and Rv3161c obtained by MALDI-TOF MS. Black highlighting indicates identical residues.

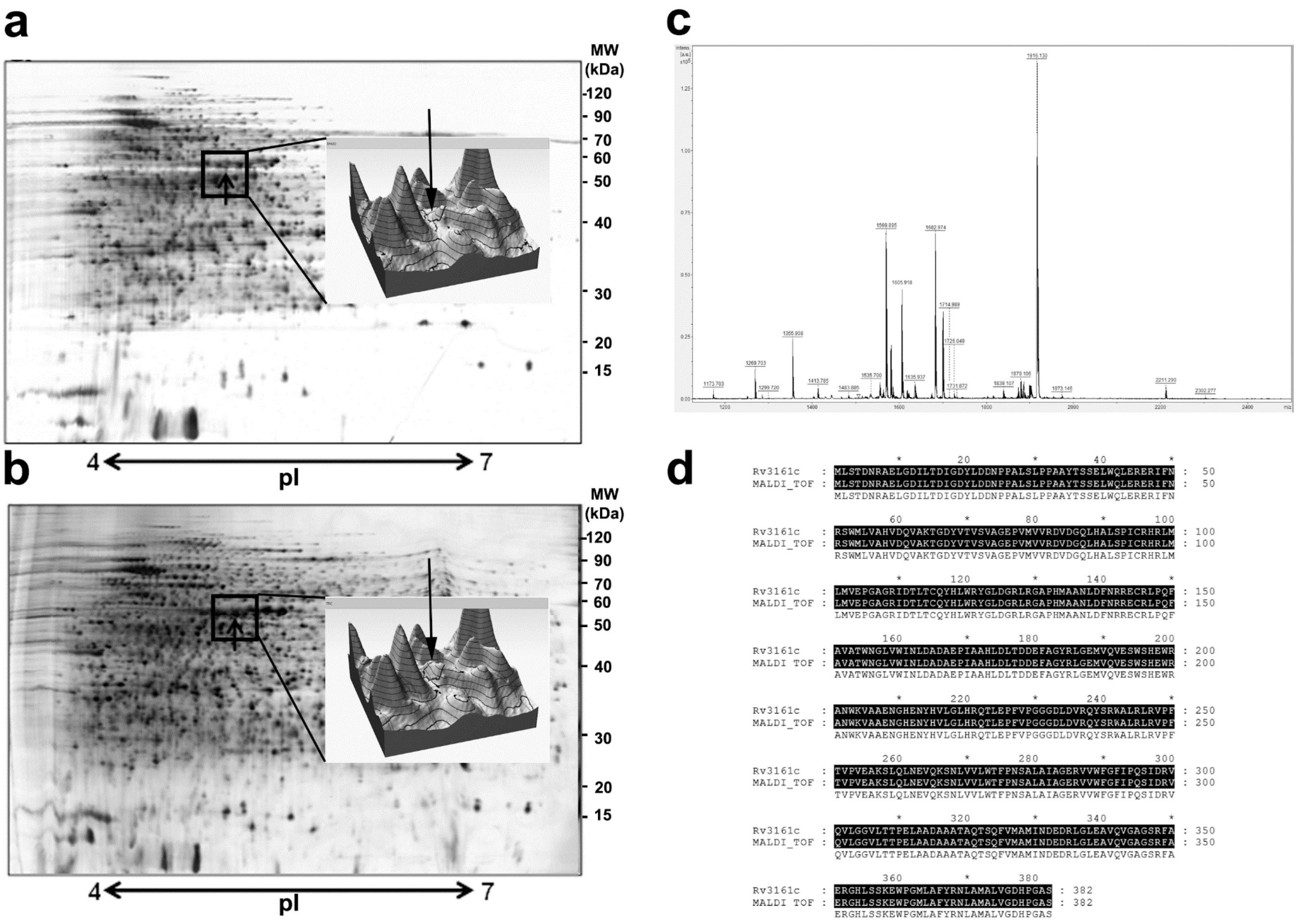

a
MW (kDa)
120
90
70
60
50
40
30
20
15
4
pI
7
b
MW (kDa)
120
90
70
60
50
40
30
20
15
4
pI
7
c
1916.130
1569.895
1682.974
1605.918
1714.989
1355.908
1269.703
1725.049
1200
1400
1600
1800
2000
2200
2400
d
Rv3161c : MLSTDNRAELGDILTDIGDYLDDNPPALSLPPAAYTSSELWQLERERIFN : 50
MALDI_TOF : MLSTDNRAELGDILTDIGDYLDDNPPALSLPPAAYTSSELWQLERERIFN : 50
MLSTDNRAELGDILTDIGDYLDDNPPALSLPPAAYTSSELWQLERERIFN
Rv3161c : RSWMLVAHVDQVAKTGDYVTVSVAGEPVMVVRDVDGQLHALSPICRHRLM : 100
MALDI_TOF : RSWMLVAHVDQVAKTGDYVTVSVAGEPVMVVRDVDGQLHALSPICRHRLM : 100
RSWMLVAHVDQVAKTGDYVTVSVAGEPVMVVRDVDGQLHALSPICRHRLM
Rv3161c : LMVEPGAGRIDTLTCQYHLWRYGLDGRLRGAPHMAANLDFNRRECRLPQF : 150
MALDI_TOF : LMVEPGAGRIDTLTCQYHLWRYGLDGRLRGAPHMAANLDFNRRECRLPQF : 150
LMVEPGAGRIDTLTCQYHLWRYGLDGRLRGAPHMAANLDFNRRECRLPQF
Rv3161c : AVATWNGLVWINLDADAEPIAAHLDLTDDEFAGYRLGEMVQVESWSHEWR : 200
MALDI TOF : AVATWNGLVWINLDADAEPIAAHLDLTDDEFAGYRLGEMVQVESWSHEWR : 200
AVATWNGLVWINLDADAEPIAAHLDLTDDEFAGYRLGEMVQVESWSHEWR
Rv3161c : ANWKVAAENGHENYHVLGLHRQTLEPFVPGGGDLDVRQYSRWALRLRVPF : 250
MALDI_TOF : ANWKVAAENGHENYHVLGLHRQTLEPFVPGGGDLDVRQYSRWALRLRVPF : 250
ANWKVAAENGHENYHVLGLHRQTLEPFVPGGGDLDVRQYSRWALRLRVPF
Rv3161c : TVPVEAKSLQLNEVQKSNLVVLWTFPNSALAIAGERVVWFGFIPQSIDRV : 300
MALDI_TOF : TVPVEAKSLQLNEVQKSNLVVLWTFPNSALAIAGERVVWFGFIPQSIDRV : 300
TVPVEAKSLQLNEVQKSNLVVLWTFPNSALAIAGERVVWFGFIPQSIDRV
Rv3161c : QVLGGVLTTPELAADAAATAQTSQFVMAMINDEDRLGLEAVQVGAGSRFA : 350
MALDI_TOF : QVLGGVLTTPELAADAAATAQTSQFVMAMINDEDRLGLEAVQVGAGSRFA : 350
QVLGGVLTTPELAADAAATAQTSQFVMAMINDEDRLGLEAVQVGAGSRFA
Rv3161c : ERGHLSSKEWPGMLAFYRNLAMALVGDHPGAS : 382
MALDI TOF : ERGHLSSKEWPGMLAFYRNLAMALVGDHPGAS : 382
ERGHLSSKEWPGMLAFYRNLAMALVGDHPGAS

References

1. Stover, C.K. *et al.* New use of BCG for recombinant vaccines. *Nature* **351**, 456-60 (1991).
2. Bardarov, S. *et al.* Specialized transduction: an efficient method for generating marked and unmarked targeted gene disruptions in Mycobacterium tuberculosis, M. bovis BCG and M. smegmatis. *Microbiology* **148**, 3007-17 (2002).
3. Jain, P. *et al.* Specialized transduction designed for precise high-throughput unmarked deletions in Mycobacterium tuberculosis. *MBio* **5**, e01245-14 (2014).
